# COVID-19 positivity associated with traumatic stress response to childbirth and no visitors and infant separation in the hospital

**DOI:** 10.1038/s41598-021-92985-4

**Published:** 2021-06-29

**Authors:** Gus A. Mayopoulos, Tsachi Ein-Dor, Kevin G. Li, Sabrina J. Chan, Sharon Dekel

**Affiliations:** 1 Department of Psychiatry, Massachusetts General Hospital, Boston, MA USA; 2 School of Psychology, Interdisciplinary Center (IDC), Herzliya, Israel; 3 Department of Psychiatry, Harvard Medical School, Boston, MA USA; 4 Present Address: Department of Counseling and Clinical Psychology, Teachers College, Columbia University, New York, NY USA

**Keywords:** Psychology, Health care

## Abstract

As the novel coronavirus (COVID-19) has spread globally, a significant portion of pregnant and delivering women were infected with COVID-19. While emerging studies examined birth outcomes in COVID-19 positive women, knowledge of the psychological experience of childbirth and maternal wellness remains lacking. This matched-control survey-based study included a sample of women recruited during the first wave of the pandemic in the US who gave birth in the previous six months. Women reporting confirmed/suspected COVID-19 (n = 68) during pregnancy or childbirth were matched on background factors with women reporting COVID-19 negativity (n = 2,276). We found nearly 50% of COVID positive women endorsed acute traumatic stress symptoms at a clinical level in response to childbirth. This group was more than twice as likely to endorse acute stress and to have no visitors during maternity hospitalization than COVID negative women; they were also less likely to room-in with newborns. The COVID positive group reported higher levels of pain in delivery, lower newborn weights, and more infant admission to neonatal intensive care units. Our findings suggest COVID-19 affected populations are at increased risk for traumatic childbirth and associated risk for psychiatric morbidity. Attention to delivering women’s wellbeing is warranted during the pandemic.

## Introduction

The coronavirus (COVID-19) pandemic’s immense scope and duration has made clear the urgent need to better understand the virus’ physical and psychological impacts on vulnerable populations. From a generational health perspective, perhaps no population’s experience is more critical to understand and safeguard than that of delivering women. In the midst of a global public health crisis characterized by a potentially lethal and highly infectious virus, many non-emergency hospital-based health procedures were postponed. Nevertheless, delivering women all over the globe were among the very few populations that continued to be treated in hospital settings. With the goal of reducing infectious exposures to visitors, other patients, the community, and healthcare teams, and in the wake of uncertain and rapidly evolving situations, policies restricting visitors have been implemented to lower the number of people in labor and delivery and maternity wards.

A significant portion of women underwent childbirth when they were suspected or confirmed of the novel coronavirus disease; COVID-19 infection was reported in 9% to 15% of women giving birth in New York City in the first wave of the pandemic in the US^1–3^. Many women were likely to have been asymptomatic^2^. Some may have experienced mild and others severe physical symptoms^3^ such as fever, lymphocytopenia, and elevated C-reactive protein^4,5^, in accord with reports that pregnancy may result in acute immune changes and cause a viral illness to be more severe^6^. Labor and delivery present physical and psychological challenges during normalcy, therefore it is critical to understand the effect of COVID-19 on delivering women^7^. At present, the impact of being COVID-19 positive, confirmed or suspected, on the childbirth experience and maternal and neonatal outcomes remains not fully clear.

Emerging studies have focused largely on obstetrical and neonatal correlates of COVID-19 infection status. A body of research suggests that there is increased risk for adverse outcomes in pregnant women with COVID-19. In a recent systematic review of nine studies in China, the incidence of preterm births, low birth weight, C-section, and NICU admission were found to be higher in COVID-19 positive cases than in the general population^8^. Likewise, in a second review of 41 cases, higher rates of preterm birth and NICU admission were found in positive pregnancies in comparison with non-positive^9^, although other studies did not find differences between affected and non-affected women in maternal and neonatal outcomes^10,11^. The inclusion of studies with small samples and inappropriate control groups in the reviews above may limit findings and interpretations.

An important issue to consider is the subjective experience of childbirth for COVID-19 positive laboring women. Although childbirth is typically considered a happy event, it can become a highly stressful experience and result in acute stress responses and subsequent psychiatric morbidity, as assessed before the pandemic^12,13^. Labor pain has been regarded as the most agonizing of painful experiences^14^ and a significant minority can experience severe morbidity surrounding childbirth^15^. Relatively little is known on women’s childbirth experiences while being physically ill with a highly transmissible virus and the potential increased psychological adversity surrounding childbirth.

In addition to endorsing symptoms that may result in more complicated deliveries, women who contracted COVID-19 may have faced various degrees of social isolation surrounding childbirth to reduce virus transmission. A significant number of COVID-19-affected women may have experienced the actual childbirth and/or the critical immediate postpartum period without the emotional support provided by having close friends or family in the room with them due to hospital restrictions^16^. They may have also faced reduced contact with their infant immediately after birth and separation from the infant as part of means to minimize virus transmission risks. Continuous social support surrounding childbirth and contact with the infant can promote successful neonatal and maternal outcomes .

As the obstetric health risks and benefits in the face of a still poorly understood virus remain unclear^17^ and hospitals across the United States continue to evaluate and adjust their policies in light of recommendations from the Centers for Disease Control and Prevention (CDC) and the American College of Obstetricians and Gynecologists (ACOG), a better understanding of the childbirth experiences in COVID-19 vulnerable mothers, such as those being suspected or confirmed of infection, is warranted.

To better understand the differing experiences of childbirth among COVID-19 positive and negative women, we surveyed a large sample of women; the majority gave birth in the outbreak of the pandemic in the US. Among them were 68 women who reported suspected or confirmed COVID-19 positivity in pregnancy or childbirth. We matched this COVID-19 positive group on a wide range of background factors to a group of 2276 women who gave birth when the pandemic was prevalent in their communities but who were negative for COVID-19. There have been no studies to date that examine the psychological experience of childbirth and use a comprehensive matched-control case study design to understand the impact of COVID-19 positivity on birth experiences and outcomes. We examined whether being COVID-19 confirmed or suspected is subsequently associated with psychological traumatic responses to childbirth and links to obstetrical and neonatal-related outcomes.

## Methods

### Participants

This study is part of an ongoing research project that was launched on April 2nd, 2020, in the midst of the COVID-19 pandemic in the United States, with the overarching goal of understanding the impact of COVID-19 on childbirth and maternal mental health. Women who had given birth in the last 6 months were recruited through announcements on our hospital’s research study platform as well as via social media and postpartum professional communities; they were asked to complete an anonymous Internet survey. Participants were informed that they goal of the study is understand the impact of COVID-19 on a woman’s childbirth experience and wellbeing. They were informed that their participation is voluntary, about the duration of the survey, and that they have the option to complete it on their own time. They were asked questions about demographics, recent childbirth, mental health, and motherhood and had the option to skip questionnaires that may make them feel uncomfortable. They were informed that by agreeing to complete the survey they were implying their consent to participate in the study. Therefore, all subjects who took the survey consented to the study. By beginning the survey and implying their consent, they were told that they are giving permission to securely store their responses in the study team database. Partners Healthcare (Mass General Brigham) Human Research Committee reviewed the study measures and procedures and granted exemption for this study and the study was carried out in accordance with the approved protocol.

The study sample was recruited between April 2, 2020 and October 2, 2020; 3,183 women responded to the survey (84.7% were non-Hispanic white, 8.7% Hispanic, 3.0% Black and 3.6% did not report their race). 2,517 gave birth since COVID was prevalent in their community and met study inclusion/exclusion criteria (18 years or older and giving birth to a live baby in the past six months, i.e., provided childbirth date) and 666 gave birth before COVID. For the purpose of this investigation, we studied women who gave birth since COVID and excluded 58 women who noted being COVID positive in the postpartum period and 115 who were missing COVID-19 status and/or were missing information on other variables in this study.

The final sample included a total of 2,344 participants who were on average two months postpartum (*SD* = 1.52 months). 84.8% gave birth in April or May 2020. All were part of the ongoing cross-sectional study investigation. They included 68 who reported being COVID-19 positive, suspected or confirmed, during pregnancy and/or childbirth (among them 12 were positive both in pregnancy and actual childbirth, 38 only in pregnancy, and 18 only in actual childbirth). The remaining 2276 participants who were COVID negative were matched with the COVID positive on various background factors. The groups were matched on demographic factors, primiparity, prior trauma and pregnancy complications, prior mental health, residence, and date of childbirth and survey completion.

In the final sample, the vast majority delivered a healthy baby at term (93.0%), had a vaginal delivery (71.0%), and around half (54.1%) were primiparas. The average age of participants was 31.75 years old (*SD* = 4.63). The majority were married (90.9%), had at least middle-class income (i.e., $100,000 per year, 54.3%), were employed (75.5%), and had at least a college degree (76.1%). 88.9% were non-Hispanic white, 7.8% Hispanic and 3.3% Black. Participants resided in the United States (86.6%), in Canada (3.8%), Europe (3.3%), Central/South America (1.4%), Asia (0.2%), Africa (0.2%) and 0.8% in the Caribbean and Middle East.

### Measures

Acute stress responses to childbirth were measured with the commonly used Peritraumatic Distress Inventory (PDI)^18^. The PDI is a 13-item self-report assessing various negative emotional responses (e.g., “I felt helpless”; “I thought I might die”) experienced during and immediately after a specified traumatic event on a 0 (not at all) to 4 (extremely true) scale. In this study, participants rated their responses in regard to their recent childbirth. The PDI has good psychometric properties and has been used to assess acute stress responses to childbirth in postpartum samples^19^. The PDI scores have been shown to prospectively predict the development of posttraumatic stress disorder(PTSD) following known traumas such as sexual assault and automobile accident^19,20^. To define clinically significant acute stress symptoms, we used the reported cutoff of 17, which is indicative of increased PTSD risk^20^. Reliability in the current study was high (α = 0.91).

Obstetric and infant factors in relation to recent childbirth were measured using single items. They included: gestational age, obstetric complications during labor or delivery (yes vs. no), degree of pain during labor and degree of pain during delivery (each assessed on a 5-point Likert scale), sleep deprivation (defined as less than six hours of sleep on the night before childbirth), use of pain medication (yes vs. no), use of labor induction medication (yes vs. no), delivery mode, newborn weight (lbs.), newborn biological sex, newborn neonatal intensive care unit (NICU) admission, skin-to-skin contact after delivery (yes vs. no), rooming-in (yes vs. no) and breastfeeding habits (exclusive, mixed, stopped, or never breastfeeding offered).

We also assessed for prior pregnancy complications (defined as miscarriage, stillbirth, or premature delivery), history of exposure to traumatic events with the well-validated Life Events Checklist for DSM-5 (LEC-5)^21^ pertaining to the number of events happened or witnessed (α = 0.91), and mental health problems before recent childbirth (i.e., depression, postpartum depression, anxiety, posttraumatic stress disorder). Demographic information collected concerned maternal age, education level, marital and employment status, income, race/ethnicity, and primiparity. COVID-19-related restrictions were assessed in regard to visitor policy. Participants were asked whether there were “any visitor restrictions during your hospital stay?”. Response options (no visitors, one visitor, no restrictions) were classified as “No visitors” versus “Other” and also classified in regard to no visitors during labor/delivery or postpartum stay. We also asked participants whether they were separated from their infant.

### Data analysis

To create matched groups who share similar background characteristics between COVID-19 positive and negative women, we conducted a full propensity score matching procedure using *MatchIt* R package. Full propensity score matching uses all available individuals in the data by grouping the individuals into a series of matched sets such that each set comprised at least 1 participant of the study group and often a few of the control group^22^. Next, weights are calculated for each participants and subsequent statistics use these weights. The groups were matched on demographics (maternal age, education, marital status, income, race/ethnicity), primiparity, prior mental health, prior pregnancy complications, trauma history, time since delivery, survey completion date, and place of residence; Table 1 shows the sociodemographic and childbirth-related characteristics of each group.

**Table 1:** Sociodemographic and health history by study group.

	COVID-19	
	Positive	Negative
	*n (%)/M (SD)*	*n (%)/M (SD)*
Maternal age	31.69 ± 5.03	31.78 ± 4.61
**Education**		
Bachelor’s degree or higher	58 (85%)	1,657 (76%)
No Bachelor’s degree	10 (15%)	517 (24%)
**Marital status**		
Married or domestic partnership	60 (88%)	1984 (91%)
Single	8 (12%)	190 (9%)
**Household income**		
< $20,000	3 (4%)	75 (3%)
$20,000–$99,999	19 (28%)	916 (42%)
$100,000–$300,000	40 (59%)	1084 (50%)
> $300,000	6 (9%)	99 (5%)
**Race and ethnicity**		
Non-Hispanic white	59 (87%)	1940 (89%)
Other	9 (13%)	234 (11%)
Primiparity	39 (57%)	1168 (54%)
Prior mental health problems	19 (28%)	691 (32%)
**Prior pregnancy complications**		
Miscarriage	25 (37%)	622 (29%)
Stillbirth	1 (1%)	26 (1%)
Premature delivery	2 (3%)	95 (4%)
Trauma history (i.e., sexual assault)	5 (7%)	226 (10%)
Postpartum months	1.71 ± 1.35	1.93 ± 1.49
**Place of residence**		
United States of America	54 (80%)	1891 (88%)
Canada	5 (7%)	80 (4%)
United Kingdom	1 (1%)	31 (1%)
Europe	3 (4%)	38 (2%)
Oceania (Australia/New Zealand)	2 (3%)	84 (4%)
Africa	–	4 (0.1%)
Middle East	–	8 (0.3%)
Asia	–	5 (0.2%)
Central/South America	3 (4%)	6 (0.2%)
Caribbean	–	6 (0.2%)

Premature delivery based on gestation age < 37; postpartum months refer to time since delivery and survey completion. Variables listed were used in the statistical matching procedure.

Following the matching procedure, we compared groups in obstetrical factors (sleep deprivation, pain in labor and birth, birth complications, medication for induction and pain, and mode of delivery), infant-related factors (gestational age, NICU admission, weight and sex, breastfeeding, rooming in, and skin-to-skin), COVID-19-related restrictions (separation from newborn and no visitors during delivery hospitalization), and psychological experience of birth, namely, acute stress responses in birth. Kolmogorov–Smirnov and Shapiro–Wilk tests were conducted to assess normality in quantitative outcome scores. Based on these tests, weighted independent t-tests were used to estimate group differences (0 = COVID negative, 1 = COVID positive) in quantitative measures, and weighted logistic and/or multinominal regression for estimating differences in categorical measures. All analyses were conducted in R^23^.

## Results

### COVID-19 positive birth-related outcomes

Percentages of birth-related outcomes are presented in Table 2; mean differences are presented in Table 3.

**Table 2:** Differences by study group in the percentage of childbirth-related outcomes (weighted).

	COVID-19					
	Positive		Negative		*z*	*OR (95% CI)*
	*%*	*n*	*%*	*n*		
Sleep deprivation	73.53	50	59.14	1346	2.34*	1.92 (1.13, 3.40)
Obstetric complications	30.88	21	23.59	537	1.39	1.44 (0.84, 2.41)
Medication for induction	50.00	34	57.86	1317	−1.29	0.73 (0.45, 1.18)
Medication for pain	75.00	51	83.48	1900	−1.83	0.59 (0.35, 1.07)
**Mode of delivery**					4.73	
Natural	19.12	13	18.23	415		1.05 (0.53, 1.08)
Vaginal	54.41	37	43.45	989		1.55 (0.93, 2.61)
Assisted	5.88	4	6.90	157		0.84 (0.22, 2.31)
Planned cesarean	8.82	6	10.72	244		0.81 (0.28, 1.88)
Unplanned cesarean	11.76	8	20.69	471		0.51 (0.21, 1.08)
NICU admission	16.18	11	8.66	197	2.10*	2.03 (1.01, 3.79)
Infant’s sex (boys)	51.47	35	47.80	1088	−0.02	0.99 (0.61, 1.62)
**Breastfeeding**					8.98*	
Exclusive	69.12	47	57.64	1312		1.64 (0.96, 2.92)
Breastfeeding + supplement	16.18	11	30.68	698		0.44* (0.21, 0.85)
Stopped	8.82	6	9.01	205		0.98 (0.34, 2.29)
No breastfeeding	5.88	4	2.50	57		2.43 (0.62, 6.88)
Rooming in	85.29	58	93.98	2139	−2.80**	0.37 (0.22, 0.75)
Skin-to-skin contact	79.41	54	90.64	2063	−2.99**	0.40 (0.20, 1.32)
Separation from infant	14.71	10	0.00	0	Inf	Inf
No visitors during hospital stay	33.82	23	16.12	367	3.72***	2.65 (1.56, 4.40)
Acute stress response to childbirth	48.53	33	26.27	598	3.93***	2.64 (1.62, 4.30)

Acute stress was defined based on score on the Peritraumatic Distress Inventory (PDI(≥ 17).

*OR* odd ratios, *95% CI* 95% confidence interval, *NICU* neonatal intensive care unit, *Inf*  infinity.

* *p* < .05, ** *p* < .01, *** *p* < .001.

**Table 3:** Differences by study group in the mean level of childbirth-related outcomes.

	COVID-19					
	Positive		Negative		*t*	Hedges’ g (95% CI)
	*M*	*SD*	*M*	*SD*		
Pain in labor	3.43	1.16	3.29	1.26	0.88	0.11 (−0.13, 0.35)
Pain in delivery	2.75	1.32	2.41	1.33	2.04*	0.26 (0.01, 0.50)
Gestational week	38.56	2.43	38.76	1.74	−0.92	−0.11 (−0.36, 0.13)
Infant’s weight	7.10	1.30	7.62	1.11	−2.17*	−0.46 (−0.71, −0.22)

Pain in labor refers to pain during the labor phase of childbirth.

*95% CI* 95% confidence interval.

* *p* < .05.

### Obstetrical-related factors

COVID-19 positive women reported significantly greater pain in delivery (weak-to-moderate in effect size) and more prevalent sleep deprivation than COVID-19 negative women. No group differences were found in pain in labor, medical complications, medication for induction and/or pain, or mode of delivery.

### Infant-related factors

A higher percentage of babies of COVID-19 positive women were admitted to the newborn intensive unit of care (*Odds Ratio, OR* = 2.03). In addition, COVID-19 positive women gave birth to infants with lower (yet normal) weights than COVID-19 negative women. Fewer COVID-19 positive women were with their newborn in the room during their stay at the hospital (*OR* = 0.37; all because of the mother’s COVID) or had skin-to-skin contact [*OR* = 0.40; 46.30% because of the mother’s COVID and 9.25% because the newborn’s COVID (*n* = 5 babies were COVID positive); the reason for no skin-to-skin contact for the rest (44.45%) was not reported] than COVID-19 negative women. In addition, fewer COVID-19 positive women engaged in breastfeeding with supplements than COVID-19 negative women (*OR* = 0.44). No group differences were found in gestational age and infant sex.

### COVID-19 restrictions

More COVID-19 positive women were separated from their newborns and had no visitors at any time point during delivery hospitalization (56.52% in both labor and delivery, 17.39% only in labor, and 20.09% only in postpartum, 6% not reported) than COVID-19 negative women.

### Psychological experience of birth

More COVID-19 positive women had clinical levels of acute stress response to birth (≥ 17 PDI total score) than COVID-19 negative women.

## Discussion

The COVID-19 pandemic offers a rare opportunity to examine the experience of childbirth under stressful conditions. As around 385,000 women give birth each day around the world and infectious disease outbreaks continue, it is critical that we generate new knowledge to inform preparations and guidelines of perinatal care during these outbreaks.

This study is the first to examine the psychological potentially traumatic experience of childbirth and obstetric and neonatal outcomes of women who had delivered during peak infection rates of the pandemic and were suspected or confirmed to have contracted COVID-19 in pregnancy or childbirth: a population likely to have undergone labor and delivery while potentially being acutely ill with the virus.

Because COVID positivity has been linked with certain socioeconomic factors that may increase childbirth adversity, we compared women who reported being COVID-19 positive, suspected or confirmed, to women who reported not having contracted the virus but who were similar on various demographics, including mental health, trauma history, and time postpartum. This matched-control design has not been implemented in previous COVID studies and allows for the generation of knowledge regarding the influence of infection status on birth outcomes.

Our findings underscore how childbirth can become a traumatic experience for vulnerable delivering women during the novel coronavirus pandemic. Here we document that nearly 50% of women in the COVID positive group endorsed acute stress symptoms at a clinical level in response to childbirth. They were more than two times as likely to experience elevated acute stress than non-affected women and reported higher levels of pain during labor even though no differences were found in factors such as obstetrical complications, medication for pain, or delivery mode between COVID positive and negative groups. Traumatic childbirth can result in enduring maternal psychiatric morbidity, namely, childbirth-related PTSD (CB-PTSD) and comorbid depression, and problems in early bonding with the infant, as documented in pre-COVID samples^12,19,24^. Maternal morbidity can further undermine the child’s welfare during a critical time of child development and is a significant contributor of maternal death^15^. Hence, the high prevalence of acute stress symptoms linked with COVID positivity suggests an increase in the prevalence of subsequent maternal psychopathology and impairments of mother-infant bonding during the novel coronavirus pandemic^7^, warranting additional attention to potential mental health concerns in this vulnerable population.

There are many possible factors likely contributing towards elevated levels of clinically acute traumatic stress response to childbirth in COVID-19 positive women. There may be threats of health consequences and concerns of transmitting the virus to the infant. Endorsement of physical symptoms may amplify the traumatic nature of childbirth for those with current infection, although only a minority of women are likely to suffer from severe COVID symptoms.

We document exposure to salient social isolation surrounding childbirth associated with COVID-19 positivity, and it could be speculated that these stressors also contribute to traumatic stress in response to birth. While ample studies support the importance of support person in birth^25^, nearly 40% of the COVID positive group had no visitors at some point during their delivery hospitalization stay, and the majority reported no visitors in the actual birth. This group was more than two times as likely not to be permitted a support person to accompany them than women negative for COVID-19. These findings suggest that hospital policies enforcing visitor restrictions were frequently implemented with delivering women suspected or confirmed of the infection.

Lack of visitors in childbirth can be seen in contrast to continuous interpersonal support in labor and delivery. Presence of a support person has been documented before the pandemic as a factor which improves obstetrical and neonatal outcomes and reduces negative birth perceptions^25,26^. In the writing of this work, visitor prohibitions have been largely lifted in maternity wards. However, those delivering who test positive for COVID-19 may still face social isolation and not be allowed visitors during their maternity hospitalization stay.

The COVID positive group was more likely to experience various degrees of physical separation from their newborn as evident in lack of immediate skin-to-skin and rooming-in. This group was also less likely to engage in partial breastfeeding. The newborns of COVID-19 positive women were two times as likely to be admitted to the NICU. The rate of 16% of NICU admission of newborns of COVID positive women is higher than expected NICU admission based on pre-COVID samples^27^ and accords with other COVID studies^9^. Physical contact and proximity in the hours of life has known benefits for maternal and infant health. It promotes mother-infant bonding and breastfeeding and lower the odds of maternal psychological morbidity^28,29^ and this in turn may contribute along with other factors to increased acute maternal stress surrounding childbirth in COVID positive women.

The documented infant separation in the immediate postpartum may suggest that COVID-19 positivity is associated with health complications in the newborn, although infants in the COVID group had lower yet normal weight and no differences were found in gestational age. It may in part reflect hospital precaution efforts to separate mother and newborn because of the mother’s COVID status or the infant’s. Although here we classified COVID positivity if during pregnancy and/or childbirth, we document that as much as 40% of women reported not rooming-in with their infant due to COVID. Lack of support person available to assist the ill mother with the infant due to visitor restrictions may have also contributed to the separation. In the writing of this manuscript, the CDC has updated its guidance and largely recommends rooming-in for a COVID-positive mother and her newborn and also breastfeeding while weighing several considerations; the guidance acknowledges that the decision process should be made respecting the women’s autonomy^30,31^.

This study’s findings may be useful in informing clinical policies during the COVID-19 pandemic. While much attention has been paid to the physical symptoms in mothers with COVID-19, our study emphasizes the importance of considering mothers’ psychological wellness. The findings suggest that increased awareness should be given in labor and delivery and postpartum units to the psychological symptoms surrounding childbirth that may arise in women who are sick with or suspected of having the virus; additionally, the potential emotional liability of not permitting a support person during hospitalization and separation from the infant should be noted. While routine screening for traumatic childbirth and risk for CB-PTSD does not exist in postpartum hospital units, our study suggests that assessment of women at high risk for acute stress responses such as delivering women who are COVID-19 positive is warranted during the pandemic. Ongoing monitoring of mental health symptoms in this high risk group after hospital discharge is important as those with stable symptoms in accord with routine care are likely to be quickly discharged and face social isolation during the postpartum period, which is considered a time of heightened psychological vulnerability^32,33^.

Shortcomings of this study include reliance on anonymous self-report measures that allowed for conducting a study swiftly during the initial heights of the pandemic but not for inclusion of patients’ medical records. We rely on respondents accurately reporting their COVID-19 infection status, and their receiving accurate information from COVID-19 testing protocols at the hospitals where they delivered. Additionally, we do not have information on the severity of respondents’ COVID-19 symptoms, only their infection status. As we did not ask participants for the names of the hospitals where they delivered, we cannot account for the precise visitor restriction policies each woman experienced and the degree to which they may or may not have influenced maternal wellness. We also cannot rule out the possibility that differences in outcomes between the groups are due to other factors that were not measured , such as mothers’ levels of anxiety regarding the health consequences of COVID-19 infection for themselves or their newborns, or underlying prior health conditions of the mother which may have impacted maternal and neonatal postpartum outcomes. While we used a well-validated measure to assess acute stress which has correspondence with clinician assessments, we did not include diagnostic measures and retrospective reports could be prone to recall bias.

It is important to consider that this convenient Internet-based sample likely introduces a bias towards white women from better-resourced socioeconomic groups, as women without access to the Internet would have been unable to participate. Additionally, the rates of COVID-19 positivity were relatively small and this may limit study generalization. While our matched-groups design reduces the possibility that differences between COVID-19 positive and negative women is due to socioeconomic (SES) status differences, it is important to note that COVID-19 has disproportionately impacted individuals with fewer SES resources, and so it is possible that the effects we observe in our sample are even more pronounced for women from lower-SES backgrounds who may have fewer support persons and resources to rely on. Finally, our findings do not permit making clear-cut conclusions on the causal factors that result in increased acute stress in COVID-19 positive women. COVID positivity referred to infection status in pregnancy or childbirth, and we did not compare study groups on the type of no visitor (in childbirth versus during postpartum hospital stay). Therefore additional investigations are warranted to better clarify the impact of COVID positivity during actual childbirth on birth experiences and outcomes.

## Conclusion

In conclusion, we find that confirmed or suspected COVID-19 positive women experience increased psychological morbidity surrounding childbirth compared to delivering women without COVID-19. We find that COVID-19 positive women are more likely to not have a support person and be separated from their infant, in part due to their COVID status. They experience increased levels of pain during delivery and give birth to newborns that are more likely to be admitted to the NICU. We theorize there could have been multiple unique stressors that increased psychological morbidity for these mothers, such as symptoms of physical illness, elevated anxiety about the impact of COVID-19 on the health of the newborn, as well as possible contextual factors, such as social isolation due to visitor restrictions or separation from the newborn. As hospitals around the world continue to update their delivery protocols for COVID-19 positive women and determine risks and benefits of visitor restriction and separation of mother-infant policies, more research is needed to optimize maternal care during these unprecedented times.
